# Roles of Calcium Regulating MicroRNAs in Cardiac Ischemia-Reperfusion Injury

**DOI:** 10.3390/cells3030899

**Published:** 2014-09-11

**Authors:** Eunhyun Choi, Min-Ji Cha, Ki-Chul Hwang

**Affiliations:** 1 Institute for Bio-Medical Convergence, College of Medicine, Catholic Kwandong University, Gangneung-si, Gangwon-do 210-701, Korea; E-Mails: ehchoi@ish.or.kr (E.C.); mjcha@yuhs.ac (M.-J.C.); 2 Catholic Kwandong University International St. Mary’s Hospital, Incheon Metropolitan City 404-834, Korea

**Keywords:** microRNA, calcium, Ca^2+^, myocardial ischemia-reperfusion injury, heart

## Abstract

Cardiac Ca^2+^ cycling and signaling are closely associated with cardiac function. Changes in cellular Ca^2+^ homeostasis may lead to aberrant cardiac rhythm and may play a critical role in the pathogenesis of cardiac diseases, due to their exacerbation of heart failure. MicroRNAs (miRNAs) play a key role in the regulation of gene expression at the post-transcriptional level and participate in regulating diverse biological processes. The emerging evidence indicates that the expression profiles of miRNAs vary among human diseases, including cardiovascular diseases. Cardiac Ca^2+^-handling and signaling proteins are also regulated by miRNAs. Given the relationship between cardiac Ca^2+^ homeostasis and signaling and miRNA, Ca^2+^-related miRNAs may serve as therapeutic targets during the treatment of heart failure. In this review, we summarize the knowledge currently available regarding the role of Ca^2+^ in cardiac function, as well as changes in Ca^2+^ cycling and homeostasis and the handling of these processes by miRNAs during cardiac ischemia-reperfusion injury.

## 1. Introduction

Myocardial ischemia-reperfusion (I/R) injury is one of the leading causes of heart failure, and the signs and symptoms of I/R injury are characterized by an insufficient supply of oxygen and nutrients to the body; slowed, asynchronous contraction; and impaired relaxation of the heart muscle [1,2]. The key regulator of cardiac function is intracellular calcium (Ca^2+^), as Ca^2+^ acts as a second messenger and plays an important role in regulating cardiac physiology and pathophysiology. Altered expression and activity of Ca^2+^-related proteins are associated with cardiac dysfunction, which is observed in many patients with heart failure [3,4].

Recently, endogenous small noncoding RNAs known as microRNAs (miRNAs) have been identified as novel regulators of gene expression at the post-transcriptional level and regulate several cellular processes, such as cell proliferation, differentiation, and apoptosis [5,6]. The biogenesis of miRNAs begin with primary miRNAs (pri-miRNAs), which transcript miRNA gene by RNA polymerase II, and have hundreds to thousands of nucleotides and single or multiple stem-loop structures with a 5' cap and a poly(A) tail. The pri-miRNAs are cleaved by the microprocessor complex, composed of RNase-III endonuclease Drosha, RNA-binding protein (RBP) DGCR8, and other cofactors, which produce 70–100-nucleotide-long hairpin-shaped precursor miRNAs (pre-miRNAs). The pre-miRNAs are exported to the cytoplasm by the nuclear export protein exportin-5, and then they are finally trimmed by another RNase-III ribonuclease Dicer, generating a mature miRNA duplex that is approximately 21 nucleotides long. Finally, only one strand (a guide strand) of mature miRNA duplex is loaded to the RNA-induced silencing complex (RISC), whereas the other strand (the passenger strand) is degraded. The guide strand is the functional strand of the mature miRNAs and negatively regulates gene expression either by inhibiting mRNA translation or inducing mRNA degradation, which results from the complete or incomplete binding to the 3' untranslated region (3' UTR) of target mRNAs [7,8,9,10].

Recently, a number of miRNAs have been discovered; however a small portion of miRNAs are known to be expressed in the heart and an even smaller portion of miRNAs have been identified to be related to cardiac Ca^2+^ homeostasis [11]. Changes in the expression profiles of miRNAs are linked to several diseases, including cardiovascular disease, cancer, and diabetes [12,13,14]. Given the relationship between intracellular Ca^2+^ homeostasis and miRNAs during the regulation of cardiac function, the ability of miRNAs to regulate Ca^2+^-handling proteins may be an important therapeutic target in the treatment of heart disease [1,14,15,16]. Among the heart-expressed miRNAs, in this review, we focused on miRNAs related to the intracellular Ca^2+^ homeostasis and Ca^2+^ regulation within myocardial I/R injury.

## 2. Role of Calcium in Cardiac Function

The maintenance of intracellular Ca^2+^ homeostasis in the heart is essential for many aspects of cardiac function, including cardiac excitation, contraction, and relaxation. Cardiac excitation-contraction coupling (EC-coupling) is associated with Ca^2+^-induced Ca^2+^ release (CICR) [17]. Transmembrane and intracellular calcium channels and transporters participate in the movement of Ca^2+^. Cardiac contraction is initiated by membrane depolarization, which leads to the activation of voltage-gated L-type Ca^2+^ channels (LTCCs) and a subsequent increase in intracellular Ca^2+^ concentration due to the influx of extracellular Ca^2+^ [18]. This Ca^2+^ influx prompts a substantial release of Ca^2+^ from the sarcoplasmic reticulum (SR) into the cytoplasm through ryanodine receptor (RyR) channels. Cytosolic Ca^2+^ binds to Ca^2+^-sensitive proteins, such as troponin, myosin, and actin, and subsequently initiates muscle contraction. During cardiac relaxation, Ca^2+^ is released from the myofilaments and pumped back into the SR via the SR Ca^2+^-ATPase (SERCA2a) and is then transported to the extracellular space through the sarcolemmal Na^+^/Ca^2+^ exchanger (NCX) [4,19].

**Figure 1:**
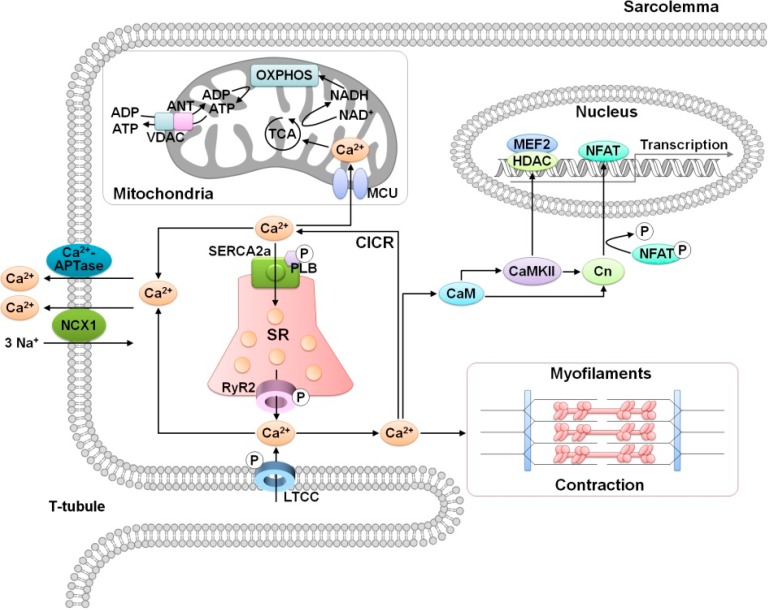
Cardiac Ca^2+^-handling and Ca^2+^-mediated signaling. LTCC, L-type Ca^2+^ channel; NCX, Na^+^/Ca^2+^ exchanger; CaM, calmodulin; CaMKII, Ca^2+^/calmodulin-dependent protein kinase II; Cn, calcineurin; RyR2, ryanodine receptor type-2; SERCA2a, sarcoplasmic reticulum Ca^2+^-ATPase; SR, sarcoplasmic reticulum; PLB, phospholamban; CICR, Ca^2+^-induced Ca^2+^ release; MCU, mitochondrial Ca^2+^ uniporter; ANT, adenine nucleotide transporter; VDAC, voltage-dependent anion channel; TCA, tricarboxylic acid; OXPHOS, oxidative phosphorylation; HDAC, histone deacetylase; NFAT, nuclear factor of activated T-cells; MEF2, myocyte enhancer factor-2; P, phosphorylation.

Another important role of Ca^2+^ is the maintenance of cardiac cellular energy homeostasis. ATP is a key energy source in cardiac excitation and contraction, and the synthesis of ATP is tightly linked to oxidative phosphorylation in mitochondria [20,21]. The mitochondrial calcium uniporter (MCU) contributes to Ca^2+^ uptake in the mitochondria from the cytosol, which activates TCA cycle enzymes and then creates the electrochemical gradient. The TCA cycle generates electrons during the conversion of NADH to NAD^+^, and these electrons are transferred through complexes I–IV of the electron transport chain (ETC). As a result of these processes, ATP is produced and released from the matrix of the mitochondrial inner membrane to the cytosol through two proteins, the adenine nucleotide transporter (ANT) on the mitochondrial inner membrane and the voltage-dependent anion channel (VDAC) on the mitochondrial outer membrane [3,22]. Therefore, cellular Ca^2+^ homeostasis plays a critical role in regulating both cardiac function and mitochondrial ATP production (Figure 1).

## 3. MicroRNA Regulation of Calcium Signaling in Myocardial Ischemia-Reperfusion Injury

Recent research has demonstrated that miRNAs participate in cardiovascular development, function, and diseases. Several miRNAs either exert cardioprotective effects or play a role in the pathogenesis of diseases [23,24], and the expression profiles of miRNAs are dynamically different during various diseases states [15,25,26]. Thus, the regulation of disease-mediated miRNAs is related to the pathophysiological mechanisms [27]. A number of miRNAs associated with cardiac I/R injury have been discovered; however, little is known regarding the detailed mechanisms by which they perform their functions. Nevertheless, miRNAs mediating cardiovascular disease, including I/R injury, have potential as therapeutic targets in disease management [28].

In this section, we focus on cardiac I/R injury, which is one of the leading causes of heart failure, cardiovascular disease, and sudden death, and provide a comprehensive summary of the changes in Ca^2+^ cycling during cardiac I/R injury and the relationship between Ca^2+^-regulating miRNAs and cardiac function.

### 3.1. Altered Ca^2+^ Homeostasis During Cardiac Ischemia-Reperfusion Injury

**Figure 2:**
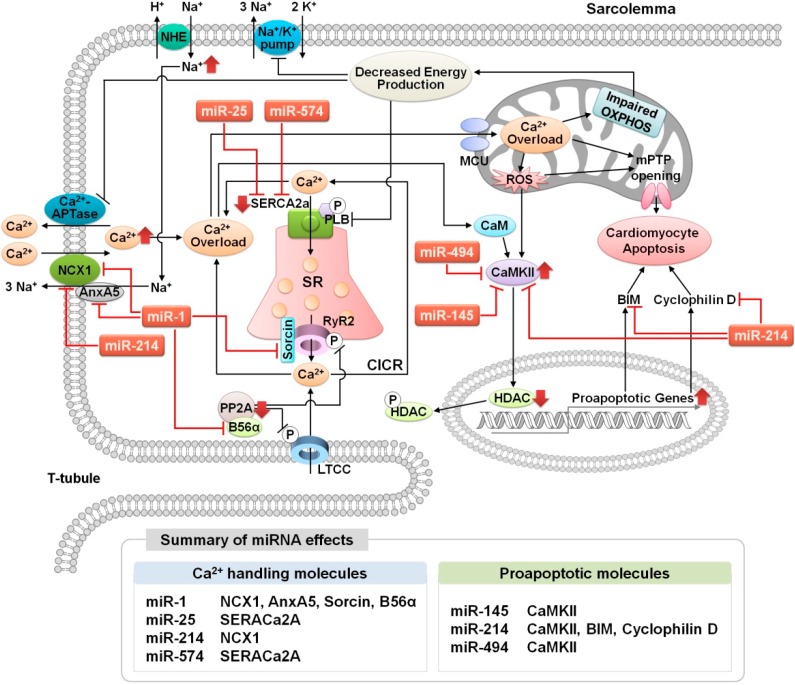
Cardiac Ca^2+^ protein-regulated microRNA during ischemia-reperfusion injury. LTCC, L-type Ca^2+^ channel; NCX, Na^+^/Ca^2+^ exchanger; AnxA5, annexin A5; NHE, Na^+^/H^+^ exchanger; CaM, calmodulin; CaMKII, Ca^2+^/calmodulin-dependent protein kinase II; RyR2, ryanodine receptor type-2; SERCA2a, sarcoplasmic reticulum Ca^2+^-ATPase; SR, sarcoplasmic reticulum; PLB, phospholamban; CICR, Ca^2+^-induced Ca2+ release; MCU, mitochondrial Ca^2+^ uniporter; ROS, reactive oxygen species; CyP-D, cyclophilin D; mPTP, mitochondrial permeability transition pore; OXPHOS, oxidative phosphorylation; HDAC, histone deacetylase; P, phosphorylation.

Cardiac I/R injury is directly linked to cardiac dysfunction and results in an imbalance in Ca^2+^ homeostasis [29,30]. I/R-injured cardiomyocytes are characterized by increased Ca^2+^ concentrations in both the cytosol and the mitochondria, which are associated with abnormal cardiac function and damage to cardiac tissues [31,32]. During ischemia, a decrease in O_2_ levels inhibits oxidative phosphorylation, which subsequently reduces ATP synthesis. The accumulation of lactate and the acceleration of acidification via anaerobic glycolysis inhibit the Na^+^/H^+^ exchanger (NHE) and the Na^+^/K^+^-ATPase, which triggers Na^+^ overload and a subsequent rise in Ca^2+^ concentration in the cytosol via the NCX, as well as abnormal activity of the Ca^2+^-ATPase. In addition, the pH of the cytosol is acidic [33,34]. Paradoxically, reperfusion is necessary to enhance the functioning of the ischemic heart; however, it also exacerbates cardiac damage due to myocardial cell death [35]. During reperfusion, mitochondrial membrane potential (ΔΨ_m_) are rapidly reestablished, leading to uncoupling of oxidative phosphorylation and a sustained rise in Ca^2+^ uptake into the mitochondria and the generation of reactive oxygen species (ROS). Ca^2+^ and ROS, as well as the restoration of neutral pH in the cytosol, accelerate the opening of the mitochondrial permeability transition pore (mPTP), which subsequently results in a significant loss of ATP and causes mitochondria-mediated apoptosis [36,37] (Figure 2).

### 3.2. MicroRNA Regulation of Ca^2+^ Signaling During Cardiac Ischemia-Reperfusion Injury

Cardiac I/R injury leads to an increase in intracellular Ca^2+^ concentration, and these Ca^2+^ ions bind to and elevate the activity of calmodulin (CaM), which activates calcineurin (Cn), a downstream molecule of both CaM and Ca^2+^/calmodulin-dependent protein kinase II (CaMKII) [11,38]. Cn has phosphatase activity and dephosphorylates nuclear factor of activated T-cells (NFAT), which translocate into the nucleus from the cytosol and upregulate the expression of cardiac pathophysiology-related genes [4]. CaMKII is a serine/threonine-specific protein kinase and a critical regulator of Ca^2+^ signaling that plays a role in cardiac disease [39,40]. Four CaMKII isoforms (α, β, γ, and δ) have been identified and have different tissue distributions. In particular, CaMKIIδ is the predominant isoform in the adult mammalian heart and has two splice variants, CaMKIIδ_B_ and CaMKIIδ_C_ [41]. CaMKIIδ_B_ has an 11 amino-acid nuclear localization signal, which is absent in CaMKIIδ_C_; therefore, CaMKIIδ_B_ localizes to the nucleus, whereas CaMKIIδ_C_ localizes to the cytosol [42]. The histone deacetylase (HDAC) class II isoforms HDAC4 and HDAC5 are phosphorylated by activated CaMKII, which regulates myocyte enhancer factor-2 (MEF-2)-mediated cardiac hypertrophy-related gene expression [43,44].

Because of the importance of Ca^2+^ in regulating proteins related to cardiac function and damage, the regulation of intracellular Ca^2+^ is considered to be a therapeutic target [35]. Several strategies have been implemented to improve Ca^2+^ regulation and to attenuate Ca^2+^ overload. Phospholamban (PLB) is a major player in regulating SERCA2a activity; in addition, PLB activity is modulated by CaMKII. Dephosphorylation of PLB inhibits SERCA2a activity, thereby preventing Ca^2+^ uptake in the SR. In this context, Shintani-Ishida *et al.* used calcineurin inhibitor cyclosporin A to prevent PLB dephosphorylation [45] and Gorbe *et al.* demonstrated that B-type natriuretic peptide (BNP) elevates PLB phosphorylation via the cGMP-dependent protein kinase (PKG) pathway [46]. In an I/R heart, CaMKII activity is increased; thus Vila-Petroff *et al.* investigated the cardioprotective effects of the CaMKII inhibitor KN-93 or the CaMKII inhibitory peptide AIP. In addition, KB-R7943, which inhibited the reverse mode NCX, showed outcomes similar to CaMKII inhibition [47]. To reduce Ca^2+^ overload, Tani *et al.* suggested a contractile inhibitor using 2,3-butanedione monoxime (BDM) [48], and Dou *et al.* identified blebbistatin, a more powerful inhibitor of the actin–myosin interaction than BDM [49]. BDM and blebbistatin maintain a cardiac muscle contractile system, including cardiomyocyte and contraction inhibition and shortening. The loss of Ca^2+^ homoeostasis activates the non-lysosomal cysteine proteases calpains, which contribute to contractile dysfunction by impairing Ca^2+^ regulation, and necrotic cell death. Therefore, calpain inhibitors promote cardioprotection against I/R injury; however, some calpain inhibitors, such as leupeptin, *N*-Ac-Leu-Leu-norleucinal (ALLN or calpain inhibitor I), and MDL-29170 (calpain inhibitor III) are unsuitable for *in vivo* applications due to poor aqueous solubility [50]. Improving the water solubility of calpain inhibitor A-705253 reduces the infarct size and improves left ventricular contractility in porcine I/R model [51]. However, the effectiveness of calpain inhibitors must be clearly confirmed in more clinically relevant animal models of human diseases, and calpain selectivity must be improved. Few clinical studies of Ca^2+^ regulation have been performed, including (1) a study of nisoldipine, a vascular-selective organic calcium antagonist [52]; (2) the DATA trial with diltiazem, another calcium antagonist [53]; (3) the CASTEMI and EVLVE randomized trials using caldaret (MCC-135), an NCX inhibitor [54,55]; (4) studies using NHE inhibitors, such as the GUARDIAN or EXPEDITION studies of cariporide [56,57] and the ESCAMI trial with eniporide [58]; and (5) the J-WIND-ANP trial, which used either human atrial natriuretic peptide or nicorandil [59]. Unfortunately, the results of these clinical trials have been insignificant or have not proven the effectiveness of these inhibitors; thus, there is still a need to understand the mechanism behind the effects of cardiac Ca^2+^ on cardiac function to develop more potent and selective modulators of Ca^2+^-related molecules.

Emerging evidence in the study of disease-related miRNAs has shown that changes in dynamic miRNA expression are linked to cardiac I/R injury, and Ca^2+^ signaling molecules are also involved in changes in the expression of several miRNAs, as discussed in further detail below (Figure 2).

#### 3.2.1. miR-25

Recently, Wahlquist *et al.* reported that miR-25 leads to an impairment in Ca^2+^ uptake and exacerbates heart failure by directly interacting with SERCA2a mRNA [60]. To identify the miRNAs that control SERCA2a, the authors constructed an eGFP-SERCA2a 3' UTR reporter vector to perform a high-content screen of 875 miRNAs and compared the results to those of SERCA2a siRNA (100% inhibition). The results of this experiment demonstrate that miR-25 is perhaps the most potent inhibitor of SERCA2a and is upregulated both in patients with severe heart failure and during trans-aortic constriction (TAC)-induced heart failure in a mouse model. Overexpression of miR-25 *in vivo* using adeno-associated virus 9 (AAV9) is related to declines in both fractional shortening and left ventricular (LV) function, whereas the inhibition of miR-25 with antagomiR reduces fibrosis and hypertrophy and improves cardiac contractility via the restoration of SERCA2a. Therefore, a strategy designed to inhibit miR-25 may be a key to developing a potential treatment for heart failure.

#### 3.2.2. miR-1

MiR-1 is one of the best-characterized miRNAs in the heart, and has been linked to cardiac development, physiology, and pathophysiology, although studies of its function are still in progress [61,62,63]. In the I/R-injured heart, cardiac-enriched miR-1 exacerbates cardiac injury and apoptosis, whereas oligonucleotides modified to act against miR-1 (e.g., LNA-antimiR-1 or loss-of-function miR-1) attenuate I/R injury by restoring protein kinase C ε (PKCε) and heat shock protein 60 (HSP60), which are targeted by miR-1 [64]. Both the overexpression and suppression of miR-1 have strong effects on Ca^2+^ flux in cardiomyocytes. The serum-response factor (SRF) regulates the transcription of NCX1 and miR-1. Tritsch *et al.* reported that the SRF/miR-1 axis regulates the expression of NCX1 and annexin A5 (AnxA5), a Ca^2+^-binding protein that interacts with NCX1, and that miR-1 is directly suppressed by both NCX1 and AnxA5 [65]. Cardiomyocyte-specific tamoxifen-inducible inactivation of the SRF gene (SRF^HKO^) results in downregulation of NCX1 mRNA and primary miR-1 expression, but the translation of both NCX1 and AnxA5 is upregulated due to the downregulation of mature miR-1. Ultimately, the upregulation of NCX1 and AnxA5 restricts intracellular Ca^2+^ extrusion during heart failure.

Ali *et al.* suggested that miR-1 had an indispensable role in cardiac contractility, in which tamoxifen-induced cardiac-specific Dicer knockout mice demonstrated a significant decrease of miR-1 expression among the examined cardiac-specific miRNAs [66]. Decreases in miR-1 are correlated with increases in Sorcin, which resides on RyR2 and is a modulator of both calcium signaling and EC-coupling. Overexpression of Sorcin leads to dysregulation of Ca^2+^ signaling and deterioration of cardiac function under conditions of cardiomyopathy, whereas the siRNA-mediated knockout of Sorcin leads to the recovery of cardiac contractility in cardiac-specific Dicer knockout mice.

The PP2A regulatory subunit B56α is another target of miR-1 and miR-133, and the inhibition of PP2A’s function via miR-1 overexpression induces hyperphosphorylation and activation of RyR2, which subsequently promotes Ca^2+^ release from the SR, leading to abnormal Ca^2+^ cycling, and increased cardiac arrhythmogenesis [67,68].

#### 3.2.3. miR-145

Increases in intracellular Ca^2+^ concentration are closely associated with the activation of CaMKIIδ, which upregulates the expression of apoptotic genes. Cha *et al.* determined that miR-145 inhibits Ca^2+^ overload and Ca^2+^-related signals by targeting CaMKIIδ, and the overexpression of miR-145 protects against ROS-induced cardiomyocyte apoptosis [69].

#### 3.2.4. miR-214

MiR-214 protects cardiomyocytes susceptible to cardiac I/R injury and heart failure and is upregulated in a variety of cardiac disease models [70]. Aurora *et al.* discovered a potential role of miR-214 in I/R injury. The heart-specific miR-214 knockout mice demonstrates that heart structure, size, and function are not significantly affected by the mutation, but Ca^2+^ regulator genes are upregulated compared to wild type mice. Additionally, miR-214 knockout mice exhibit more severe cardiac injury, including a greater extent of cardiomyocyte loss, larger fibrotic regions, and impaired cardiac performance compared to wild type mice according to a left anterior descending coronary artery (LAD) model [71]. The primary Ca^2+^ pump NCX1, a proapoptotic Bcl2 family protein known as BIM, CaMKIIδ, and cyclophilin D (a major regulator of mPTP) are direct targets of miR-214. Cardiomyocytes from miR-214 knockout mice show both high levels of intracellular Ca^2+^ and Ca^2+^ overload. Therefore, miR-214 regulates Ca^2+^ homeostasis, and prevents cardiomyocyte death and protects against cardiac I/R injury by inhibiting the expression of the Ca^2+^-handling molecule NCX1, as well as CaMKIIδ, BIM, and cyclophilin D.

#### 3.2.5. miR-494

MiR-494 is one of the miRNA transcripts that is downregulated in human infarcted and murine I/R-injured heart [11]. Wang *et al.* demonstrated that cardiac-specific overexpression of miR-494 in transgenic mice heart improves the recovery of cardiac function, reduces myocardial infarction size and prevents apoptosis by targeting the proapoptotic proteins PTEN, ROCK1, and CaMKIIδ, as well as antiapoptotic proteins FGFR2 and LIF, and via the subsequent activation of AKT signaling in mitochondria [72].

#### 3.2.6. miR-574-3p

In the infarcted myocardium, both the expression and function of SERCA2 are reduced, thereby inhibiting Ca^2+^ reuptake into the SR and subsequent Ca^2+^ overload, and the expression profiles of miRNAs are altered. The results of microarray and bioinformatics analyses demonstrate that miR-574-3p, which is upregulated in the area of myocardial infarcted, is a putative candidate miRNA that targets SERCA2a, but the authors suggest that further study (as opposed to immediate retesting) is necessary to confirm the relationship between miR-574-3p and SERCA2a [73].

#### 3.2.7. miRNAs Related to Cardiomyocyte Cell Death: miR-15, miR-21, and miR-144/451 Cluster

MiR-15a/b are increased in the mice I/R injury model, and the inhibition of miR-15 (anti-miR-15 LNA) protects cardiomyocytes from hypoxia-induced cell death [74,75]. In addition, miR-21 is sensitive to ROS and protects against I/R-induced cardiac cell death by targeting programmed cell death 4 (PDCD4) [76]. The expression of the miR-144/451 cluster is regulated by GATA-4, a critical transcription factor in the heart, and the miR-144/451 cluster protects against I/R-induced cardiomyocyte apoptosis [77].

### 3.3. Circulating MicroRNAs as New Biomarkers

Circulating disease-mediated miRNAs are new biomarkers and therapeutic targets in the treatment of cardiovascular diseases [78,79]. The release mechanism, the causes of secretion and the altered expression profiles of the circulating miRNAs are still unknown. Some evidence suggests that circulating miRNAs may be released extracellularly, assisted by lipid vesicles such as exosomes, microvesicles, or apoptotic bodies that guard miRNAs against degradation by RNases [80,81]. During the I/R injury, plasma levels of miR-1, miR-133a, miR-499-5p, and cardiac-specific miR-208b rapidly increase in both rodent models and in human patients presenting with ST-elevation myocardial infarction (STEMI). In addition, circulating miR-1, miR-133a, miR-328, miR-499, and miR-208b levels also increase in patients who present with acute myocardial infarction [82,83]. Circulating miRNAs are attractive diagnostic biomarker; however, because of the low levels of miRNAs in the blood, it is necessary to develop reliable detection and analysis methods. However, the study of circulating disease-related miRNAs enables the development of the next generation of potent biomarkers and the early diagnosis and treatment of many diseases [81].

## 4. Conclusions

Recent research has provided increasing evidence that miRNAs are an important regulator of cardiac contractility and diseases, which are processes that are mediated by Ca^2+^ cycling abnormalities. Given the importance of cellular Ca^2+^ homeostasis and signaling in cardiac function and pathogenesis, a relatively small set of only a few Ca^2+^-handling miRNAs have been discovered. In addition, clinical trials of cardiac disease-related miRNAs have not yet been initiated. Further research is necessary and may help us to identify new miRNAs that serve as potent therapeutic targets treating I/R-injured heart, allowing the elucidation of more detailed mechanisms of the role of miRNA-Ca^2+^-related proteins and genes in cardiac physiology and pathophysiology. These study results should facilitate more appropriate clinical trials to confirm the therapeutic function of miRNAs and the development of new drugs to treat cardiovascular diseases.
